# Effects of Traumatic Lesions of Nerves

**Published:** 1868-12

**Authors:** 


Effects of Traumatic Lesions of Nerves.—In our last No., p. 110, we gave a brief notice of a memoir on this subject by M. Paulet. We have since received the following fuller report of the results of the investigations of M. Paulet, which we now transfer to our pages:—

M. Paulet recently read, at the Paris Surgical Society, a highly interesting memoir on the Effects of Traumatic Lesions of Nerves, in which he arrived at some remarkable conclusions. Examining in detail the results derived from experimental physiology, as reported during the last half century, he finds the general conclusion is, that a divided nerve is capable of true regeneration, and that such regeneration is a *sine qua non* of a restoration of the function lost by the division of the nerve. Next, the period of time requisite for this regeneration may occupy one or many months, according to the amount of loss of substance, or the degree of separation between the divided ends, while, when the loss of substance exceeds a certain limit, restoration will never be accomplished. But when he comes to examine recorded facts derived from clinical experience, he finds that they lead to very different conclusions to those deduced from experiment. The facts he has collected show that the reëstablishment of function takes place long prior to the periods fixed by the physiologists, and that both sensibility and motion have been reëstablished, although the loss of substance in the trunk of the nerve has never been repaired. He divides his cases into two categories—those in which there has been simple neurotomy, and those in which this has also been accompanied by excision. Of the ten examples of the first which he has collected, he finds that in four the nervous influence was restored before the possibility of the production of a cicatrix permeable to the nervous influence, while in three instances the continued separation of the extremities of the nerves was demonstrated by preparations. Among the eighteen cases of neurotomy with excision that he has collected, in some the functions were very soon reëstablished, while in others, in which this required a much longer time, so much of the nerve had been removed as to exclude all idea of its reproduction. In some cases excision of an important nerve in nowise disturbed sensation or voluntary motion. M. Paulet passes in review the various explanations that have been given of the return of nervous function, without finding any of them satisfactory, and he has instituted experiments on animals without obtaining any elucidation; nor does he believe that it is in this direction that the explanation will be found, clinical observation carefully conducted being really what is required.—*Med. Times and Gaz.*, April 4, 1868.

